# Green Tea Catechins Induce Inhibition of PTP1B Phosphatase in Breast Cancer Cells with Potent Anti-Cancer Properties: In Vitro Assay, Molecular Docking, and Dynamics Studies

**DOI:** 10.3390/antiox9121208

**Published:** 2020-11-30

**Authors:** Alicja Kuban-Jankowska, Tomasz Kostrzewa, Claudia Musial, Giampaolo Barone, Giosuè Lo-Bosco, Fabrizio Lo-Celso, Magdalena Gorska-Ponikowska

**Affiliations:** 1 Medical Chemistry Department, Medical University of Gdańsk, 80-210 Gdańsk, Poland; tomasz.kostrzewa@gumed.edu.pl (T.K.); claudia.musial@gumed.edu.pl (C.M.); 2 Department of Biological, Chemical and Pharmaceutical Sciences and Technologies, University of Palermo, 90128 Palermo, Italy; giampaolo.barone@unipa.it; 3 Department of Mathematics and Computer Science, University of Palermo, 90133 Palermo, Italy; giosue.lobosco@unipa.it; 4 The Euro-Mediterranean Institute of Science and Technology, 90139 Palermo, Italy; 5 Department of Physics and Chemistry ‘Emilio Segrè’, University of Palermo, 90128 Palermo, Italy; fabrizio.locelso@unipa.it; 6 Institute of Biomaterials and Biomolecular Systems, Department of Biophysics, University of Stuttgart, 70174 Stuttgart, Germany

**Keywords:** protein tyrosine phosphatase inhibitor, PTP1B, breast cancer, green tea catechins, epigallocatechin

## Abstract

The catechins derived from green tea possess antioxidant activity and may have a potentially anticancer effect. PTP1B is tyrosine phosphatase that is oxidative stress regulated and is involved with prooncogenic pathways leading to the formation of a.o. breast cancer. Here, we present the effect of selected green tea catechins on enzymatic activity of PTP1B phosphatase and viability of MCF-7 breast cancer cells. We showed also the computational analysis of the most effective catechin binding with a PTP1B molecule. We observed that epigallocatechin, epigallocatechin gallate, epicatechin, and epicatechin gallate may decrease enzymatic activity of PTP1B phosphatase and viability of MCF-7 cells. Conclusions: From the tested compounds, epigallocatechin and epigallocatechin gallate were the most effective inhibitors of the MCF-7 cell viability. Moreover, epigallocatechin was also the strongest inhibitor of PTP1B activity. Computational analysis allows us also to conclude that epigallocatechin is able to interact and bind to PTP1B. Our results suggest also the most predicted binding site to epigallocatechin binding to PTP1B.

## 1. Introduction

Green tea—*Camellia sinensis* L.—is a substance known for its antioxidant properties. The largest group of substances occurring in green tea are polyphenolic compounds—which have antioxidant and inflammatory properties. Green tea leaves also contain phenolic acids, amino acids, fats, and proteins and carbohydrates [1]. Among the polyphenolic biologically active compounds contained in green tea, the catechins are promising pro- and anti-oxidizing agents. Scientific research to date indicates that the number of hydroxyl groups as well as the presence of characteristic structural groups in molecules has a decisive influence on the antioxidant activity of catechins. In addition, they have the strong potential to neutralize the reactive oxygen and nitrogen species [2,3]. It has been already presented that green tea can be widely used in the prevention of breast, lung, esophagus, mouth, stomach, small intestine, colon, liver, pancreas, prostate, and mammary gland cancers [4,5,6,7].

The group of monomeric aglycons—catechins (flavan-3-ols)—belonging to the flavonoid group include epicatechin (EC), epigallocatechin (EGC), epicatechin gallate (ECG), and epigallocatechin gallate (EGCG) containing eight hydroxyl groups [8]. The chemical structure of green tea polyphenols has a significant impact on the antioxidant capacity—meta-5,7 dihydroxy structure and di-/tri-hydroxyl structure of B and D rings, as well as the presence of not less than five hydroxyl groups. The process of carcinogenesis can be initiated by the production of reactive oxygen species and the formation of oxidative stress. Research to date has shown that green tea catechins limit the production of free radicals, as well as stimulate the antioxidant process of cells before the tumor formation process begins. An interesting fact is that catechins derived from green tea are noticeable during the three stages of cancer formation—the stage of initiation, promotion, and aggression [9].

Scientific research indicates that the level of oxidative stress is affected, among others, by the enzymatic activity of protein tyrosine phosphatases, which include PTP1B—cytoplasmic phosphatase, a protein involved in cell signaling. Disorders in the action of enzymatic reactions can lead to the development of carcinogenesis, the induction of breast cancer and the metastasis process [10].

The role of protein tyrosine phosphatases (PTPs) in the formation and development of tumors was presented during the implementation of considerable scientific research. PTPs have been shown to be involved in glioblastomas, colon, lung, breast, stomach cancer, and multiple myeloma. Thus, inhibitors of PTPs (such as PTP1B phosphatase) can be potentially important in the treatment of many types of cancers, including breast cancer [11]. Phosphatase PTP1B dephosphorylates tyrosine kinases essential for the induction of breast cancer, such as HER1/EGFR, Src, JAK, and STAT, and initiates tumor formation. Overexpression and mutation of PTP1B phosphatase in breast cancer cells were already been observed [12]. Because of the key contribution of protein tyrosine phosphatases in cancer biology, they may be promising targets for the development of new anticancer diagnostic and therapeutic strategies [13,14].

Here, we present the effect of selected green tea catechins on enzymatic activity of PTP1B phosphatase and viability of MCF-7 breast cancer cells.

## 2. Materials and Methods

### 2.1. Reagents

Phosphatase PTP1B (No. SRP0215) was obtained from Sigma Aldrich, Schnelldorf, Germany. MCF-7 cell line was purchased from the European Collection of Cell Cultures (ECACC). Green tea catechins, cell media, supplements and other reagents were obtained from Sigma Aldrich.

### 2.2. Cell Line and Culture Conditions

The cells were cultured in DMEM medium supplemented with 10% fetal bovine serum, 100 μg/mL penicillin/streptomycin, and 2 mM l-glutamine. The culture was maintained at 37 °C and in an atmosphere containing 5% CO_2_. The cell culture density was kept to maximum 1 × 10^6^ cells/mL. At least every two days, the medium was replaced with the fresh one, and the cells were counted and reseeded to maintain the recommended density.

### 2.3. Cell Viability Assay (MTT Assay)

The MCF-7 cells (1 × 10^6^ cells/mL) untreated (control) or treated with solutions of catechins after the appropriate incubation time were suspended in a solution of 0.5 mg/mL (3-[4,5-dimethylthiazol-2-yl]-2,5-diphenyltetrazolium bromide) in PBS without phenol red. The 100 μL samples were incubated for 2–4 h at 37 °C in 96-well plates. When the purple precipitate was clearly visible under the microscope, 100 μL of DMSO was added to each well, and the plate with cover was left in the dark for 2–4 h. The absorbance at 540 nm was determined using a microplate reader. The experiments were performed at least three times.

### 2.4. Recombinant PTP1B Assay

Human recombinant PTP1B protein tyrosine phosphatase was obtained from Sigma-Aldrich. The solutions of the recombinant PTP was prepared in 10 mM HEPES buffer pH 7.4. The final concentration of phosphatase in reaction samples was 1.5 μg/mL (3.3 nM). PTP1B enzyme was untreated (control) or treated with a solution of green tea catechins. The assay was performed in 96-well microplates, and the final volume of each sample was 200 μL. The enzymatic activity of PTP1B was measured using 1 mM chromogenic substrate para-nitrophenyl phosphate (*p*NPP) in 10 mM HEPES buffer pH 7.4, at 37 °C. Phosphatase hydrolyzed *p*NPP to para-nitrophenol and inorganic phosphate. Para-nitrophenol is an intensely yellow colored soluble product under alkaline conditions. The increase in absorbance (due to para-nitrophenol formation) is linearly proportional to enzymic activity concentration (with excessive substrate, i.e., zero-order kinetics) and was assessed at 405 nm on a microplate reader Jupiter (Biogenet, Jozefow, Poland) using DigiRead Communication Software (Asys Hitech GmbH, Eugendorf, Austria). The experiments were performed at least three times.

### 2.5. Modelling

The structure of PTP1B was taken from the Protein Data Bank, pdb id 1SUG. The structure of the complexes between epigallocatechin and PTP1B was first modelled through molecular docking calculations, using the Autodock Vina package. The docking box, based on the ligand size and shape, was determined by Autodock Tools. Six model complexes were selected for further molecular dynamics (MD) simulations. MD simulations were performed for 150 ns, using the GROMACS 5.1.1 package. Interactions were described using an all-atoms CHARMM27 force field. The simulations for the various systems were performed using a cubic box of NaCl 150 mM in explicit TIP3P water solution. Periodic boundary conditions were applied. The force field parameter files and initial configuration for the protein were created by GROMACS utilities programs. The force field parameters of epigallochatechin have been derived from the Merck Molecular ForceField (MMFF), with Van der Waals parameters taken from the closest atom type in CHARMM22, through the SwissParam web interface. The equilibration procedure was performed in several steps, starting from an NVT simulation at 300 K with the protein heavy atom positions restrained to equilibrate the solvent around it, followed by a NPT run at 300 K and pressure at 1 bar, for a 10 ns run. After the equilibration phase, the system was run for an NVT production; the trajectory was saved at a frequency of 10 ps to evaluate dynamical and structural properties. The simulations were always checked versus the root mean square displacement (RMSD) and the energy profile. During the production runs a velocity rescaling thermostat was used for the temperature coupling, with a time coupling constant of 0.1 ps. A Parrinello–Rahman barostat was used for the pressure coupling, with relaxation constant of 1 ps. The equations of motion were integrated through the Leap-Frog algorithm, using a 2 fs time step. The values of cut-offs of the Lennard–Jones and real space part of the Coulombic interactions were set to 10 Å. The Particle Mesh Ewald (PME) summation method was used to evaluate the electrostatic interactions, with an interpolation order of 4 and 0.16 nm of FFT grid spacing. The six epigallocatechin structures (called 1–6) shown around the PTP1B protein have been selected by a clustering analysis performed by the g_cluster tool implemented in GROMACS package, following the method outlined in a cited article. Protein–ligand interactions were found by using the PLIP service. Protein pictures and manipulation were done using Maestro (Maestro, Schrödinger, LLC, New York, NY, USA, 2018, version 11.6.010) and Chimera [15].

### 2.6. Statistical Analysis

Experiments were done in triplicates, and the results are reported as mean ± standard deviation. Best fit linear regression analysis was carried out using. The data were applied to GraphPad Prism (GraphPad Software, v.4, La Jolla, CA, USA). From statistical methods, we used regression analysis. The one-way ANOVA test combined with Dunnett test were also utilized in this study. The data were expressed as means ± SD. Differences between means were considered significant for *p* < 0.05.

## 3. Results

### 3.1. Inhibitory Effect of EC, EGC, ECG, and EGCG on the Enzymatic Activity of PTP1B

**Figure 1:**
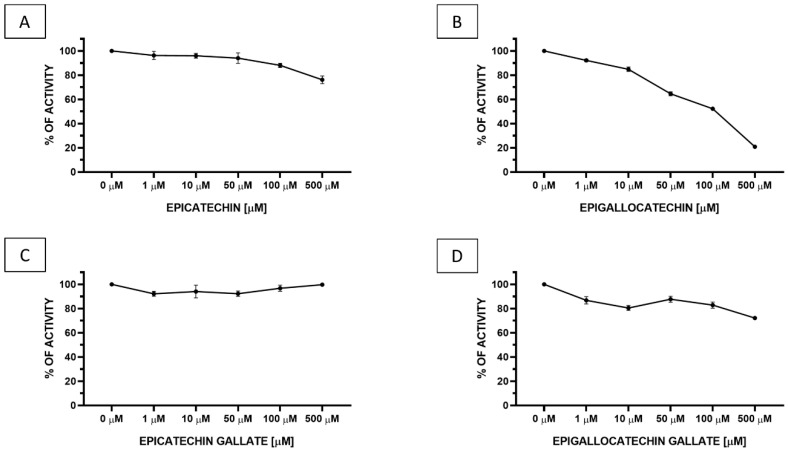
Enzymatic activity of PTP1B after 30 min of treatment with different concentrations of (**A**) epicatechin, (**B**) epigallocatechin, (**C**) epicatechin gallate, and (**D**) epigallocatechin gallate. The results were presented as a percentage of control as means ± SD (*n* = 3).

**Figure 2:**
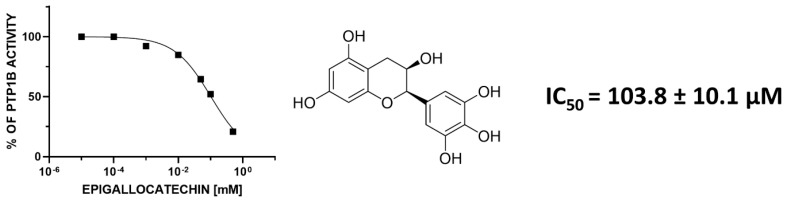
IC_50_ value for epigallocatechin as PTP1B inhibitor.

We performed an PTP1B activity assay, to determine impact of four compounds (epicatechin, epigallocatechin, epicatechin gallate, and epigallocatechin gallate) on recombinant PTP1B phosphatase. As we can observe, all of the tested compounds are able to decrease the enzymatic activity of PTP1B; however, epicatechin gallate was only slightly decreased (Figure 1). As demonstrated in Figure 1, one of four analyzed compounds, epigallocatechin, was the most effective and decreased the enzymatic activity of PTP1B phosphatase in a concentration dependent manner. We observed that incubation with 500, 100, 50, 10, and 1 μM epigallocatechin reduced activity of PTP1B to 20, 51, 65, 85, and 92%, respectively. We calculated IC_50_ for epigallocatechin against PTP1B enzymatic activity, which was equaled to 103.8 ÷ 10.1 μM (*p* < 0.00001), as demonstrated in Figure 2.

### 3.2. EC, EGC, ECG, and EGCG Effect on the Viability of MCF-7 Breast Cancer Cells

**Figure 3:**
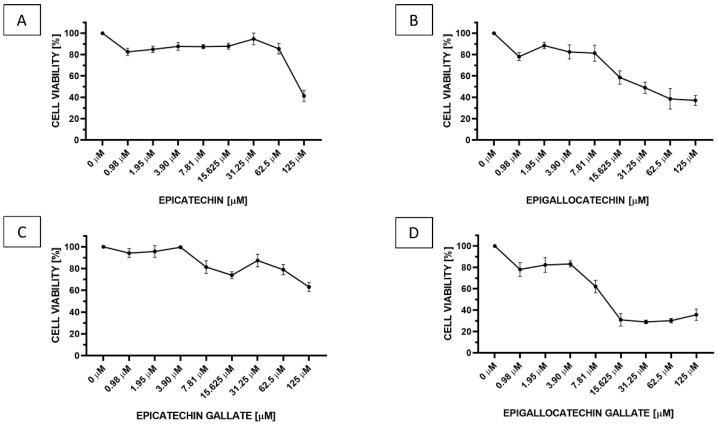
The cellular viability of MCF-7 after 24 h incubation with different concentrations of (**A**) epicatechin, (**B**) epigallocatechin, (**C**) epicatechin gallate, and (**D**) epigallocatechin gallate. The cellular viability was measured by MTT Cell Viability assay. The results were presented as a percentage of control (mean ± SD, *n* = 3).

To estimate the effect of selected catechins on breast cancer cells, the MCF-7 cells were treated with serial concentrations of epicatechin, epigallocatechin, epicatechin gallate, and epigallocatechin gallate. The cells were incubated for 24 h with 0.98 to 125 μM of selected compounds. As presented in Figure 3, the results show that all tested compounds are able to decrease the MCF-7 cell viability, and the inhibition is in a concentration dependent manner. The highest inhibitory effect on cell viability induces epigallocatechin gallate, which is the most effective starting from even 15.625 μM concentration (Figure 3D).

**Figure 4:**
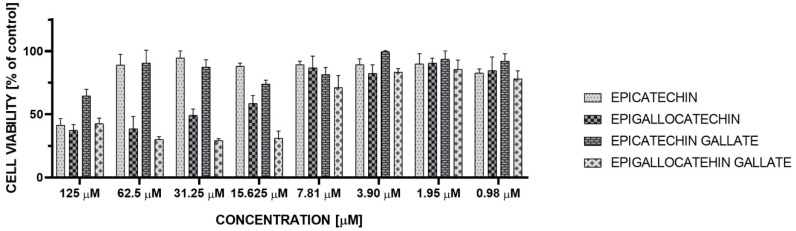
The cellular viability of MCF-7 cells after 24 h incubation according to green tea catechin’s concentration (mean ± SD, *n* = 3).

**Figure 5:**
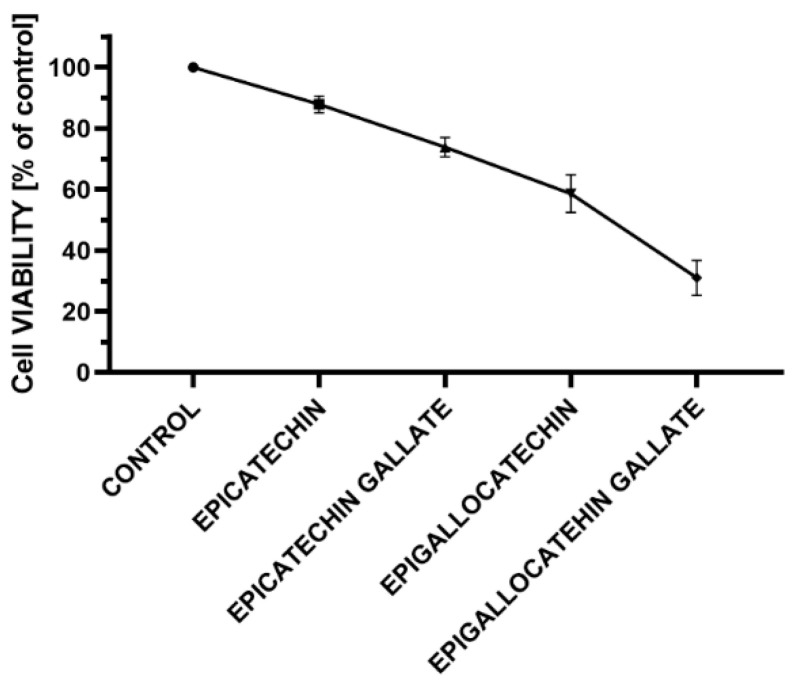
The cellular viability of MCF-7 after 24 h with 25 µM epicatechin, epicatechin gallate, epigallocatechin, and epigallocatechin gallate. The cellular viability was measured by MTT assay (mean ± SD, *n* = 3). The results were presented as a percentage of control.

The cellular viability of MCF-7 cells after 24 h incubation according to green tea catechin’s concentration is also presented in Figure 4 and Figure 5. We observed that 24 h incubation with epicatechin in concentration 125 μM reduced the MCF-7 viability to 41% (**** *p* < 0.00001). While epicatechin at the concentrations range of 0.98–62.5 μM had no significant impact on cell viability, incubation with concentrations range to 62.5–125 μM epigallocatechin reduced the MCF-7 viability to 37% (**** *p* < 0.00001), 31.25 μM to 49% (**** *p* < 0.00001), and 15.625 μM to 57% (*** *p* < 0.0001). Epigallocatechin at the concentrations range 0.98–7.81 μM had no significant impact on cell viability. Incubation with concentrations 7.81, 15.625, and 125 μM epicatechin gallate reduced the MCF-7 viability to 81% (* *p* < 0.1), 74% (** *p* < 0.001), and 65% (**** *p* < 0.00001), respectively. Other concentrations of epicatechin gallate had no significant impact on cell viability. Incubation with concentration range 0.98–125 μM epigallocatechin gallate reduced the MCF-7 cell viability to 78 (** *p* < 0.001), 85 (no significant), 83 (* *p* < 0.1), 71 (*** *p* < 0.0001), 31 (**** *p* < 0.00001), 29 (**** *p* < 0.00001), 30 (**** *p* < 0.00001), and 42% (**** *p* < 0.00001), respectively (Figure 4).

**Figure 6:**
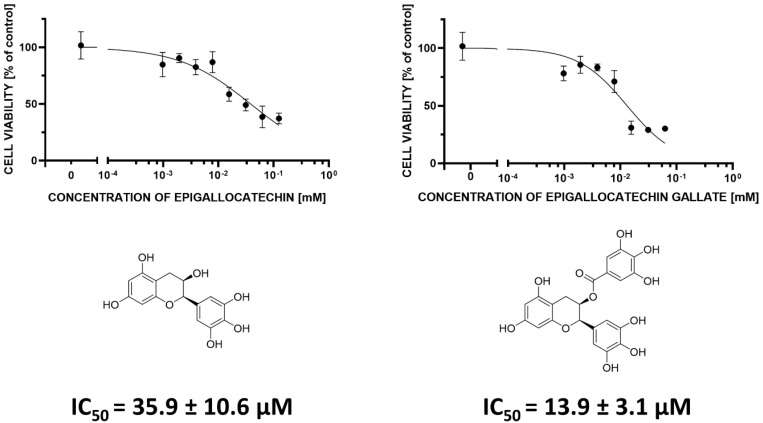
IC_50_ values for epigallocatechin and epigallocatechin gallate in MCF-7 cellular model.

We calculated IC_50_ values for two most effective compounds, epigallocatechin, and epigallocatechin gallate, in MCF-7 cellular model, in nonlinear log (inh) vs. normalised response—variable slope. The IC_50_ values are showed in Figure 6.

Comparing the inhibitory effect of tested compounds against PTP1B enzymatic activity and MCF-7 viability presented as IC_50_ values (Table 1), we can conclude that epigallocatechin is the most effective inhibitor from tested compounds both oncogenic PTP1B phosphatase and breast cancer cell viability. Both epigallocatechin and epigallocatechin gallate were able to decrease the MCF-7 cell viability with the strongest effect.

### 3.3. Docking EC, EGC, ECG, and EGCG to PTP1B

#### Molecular Modelling Studies

**Figure 7:**
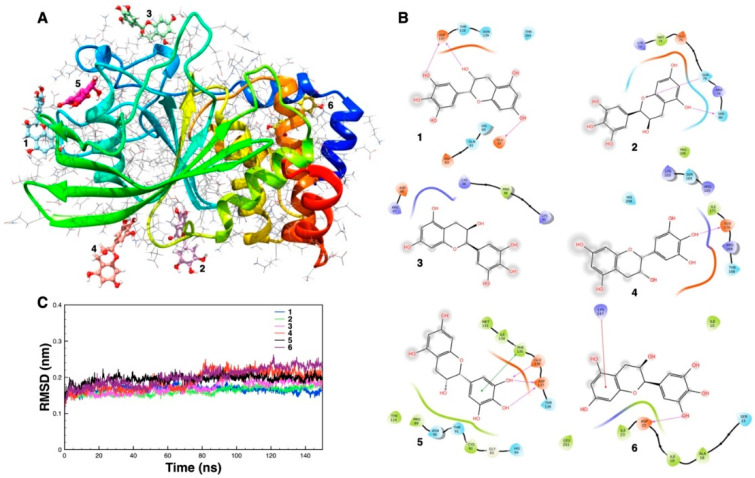
(**A**) The six different binding sites of epigallocatechin with PTP1B found obtained after six respective molecular dynamics (MD) simulations. (**B**) Two-dimensional analysis of the protein residues surrounding the epigallocatechin in each binding site. (**C**) Root mean square displacement (RMSD) plots of the six MD trajectories.

The six different binding poses of epigallocatechin with PTP1B, found at the end of 150 ns of MD simulations, are shown in Figure 7A. The 2D pictures of the residues involved in the binding of the ligand and RMSD plot, for each of the six simulations, are also reported in Figure 7B,C, respectively. Among the different binding positions, the one labeled 5 shows the larger number of H-bonds between the ligand and the protein. Moreover, it is inserted more deeply within the indicated binding pocket. These results suggest that the binding at site 5 is by far preferable compared to the other binding sites found.

## 4. Discussion

More and more scientific research is focused on the use of botanical properties, or more precisely, the bioactive ingredients contained in them, in the prevention of cancer. It is known that free radicals have a decisive influence on the formation of tumors [15]. Polyphenols, however, have antioxidant and neutralizing free radicals. Antioxidants suppress oxidation reactions caused by reactive oxygen and nitrogen free radicals. In this way, they delay and prevent cell damage [16]. Green tea, which is the subject of our research, is one of the most popular drinks consumed in the world. In addition, it contains a number of powerful antioxidants and, more specifically, polyphenols [17].

The group of polyphenols contained in green tea (*Camellia sinesis*) includes catechins and phenolic acids [18]. Catechins belong to the group of flavonoids and reveal antioxidant activities. Interesting is the fact that the amount of catechins in the infusion of green tea is influenced by factors such as growing conditions [19]. Green tea catechins are recognized as an effective preventive measure in the field of cancer. Available data indicate potential efficacy against esophageal cancer, liver cancer, prostate cancer, or breast cancer [20]. However, from a medical point of view, it should be emphasized that green tea polyphenols cannot replace oncological treatment, such as chemotherapy or radiation. Nevertheless, their effective antioxidant activity may support cancer prevention as well as support the effectiveness of chemotherapeutic agents. Indeed, in addition to chemopreventive activity, green tea catechins also exhibit anti-inflammatory as well as antimicrobial activity [21].

There are four main catechins contained in green tea: epicatechin (EC), epigallocatechin (EGC), epicatechin 3-gallate (ECG), and epigallocatechin 3-gallate (EGCG). EGCG is the most studied and widespread catechins [20]. This catechin is called the main polyphenol of *Camellia sinesis* [22]. However, in our studies, we considered all four green tea catechins. As presented in results section, we showed that EGCG were one of the most effective also in our studies.

As mentioned above, EGCG is commonly referred to as the central derived catechins from green tea. Anticancer properties EGCG is a leading research point. The available data indicate an antiproliferative effect on cells, acting against angiogenesis [23]. In addition, it is characterized by effective cell cycle arresting action through enzyme regulation as well as induction of apoptosis. The signaling pathways responsible for anti-tumor activity are mainly PI3K/AKT, MAPK, JAK/STAT [24]. In vivo research, based on the consumption of green tea, draws attention to the effective anti-cancer effect in lung cancer, stomach cancer, liver cancer, or colorectal cancer [24]. In addition, EGCG has an inhibitory effect on the growth of pancreatic cancer [25].

In addition to the chemopreventive potential, EGCG possesses also antioxidant effects [26]. This catechin is considered as a free radical scavenger. Antioxidant activity is based on limiting damage caused by oxidative stress, as well as inhibiting reactive oxygen species. Indeed, antioxidants also improve mitochondrial function [27].

However, antioxidants can also act as pro-oxidants when used in too-high concentrations [28]. The pro-oxidative effect consists in the production of hydrogen peroxide, hydroxyl radicals, and other intermediates of this process. EGCG used at physiological concentrations, from 1 to 50 µM, can produce low doses of reactive oxygen species. This mechanism is created to stimulate appropriate protective processes through the activation of signal paths [29].

The anti-cancer role of EGCG has been confirmed in various types of cancer and is still being under explored. This green tea compound role in diseases management can be attributed to its antioxidant and anti-inflammatory properties [30]. The chemopreventive effect of EGCG has also been proven in in vitro and in vivo studies in cancer stem cells. Stem cells have the ability to proliferate, i.e., maintain, a constant number of cells, as well as self-renewing. In addition, stem cells have the ability to differentiate into any type of cell. Research indicates that green tea extract, including EGCG, inhibits growth in both animal and cellular models [31].

EGCG possess also anti-inflammatory properties [32]. Inflammation is closely related to the release of reactive oxygen species (ROS) and pro-inflammatory cytokines. The inflammatory response is characterized by a large number of aggregations of immune cells at the site of inflammation. The EGCG anti-inflammatory mechanism is associated with the signal transduction process. Available data indicate that EGCG has inhibitory activity against IL-8 through airway epithelial cells [33].

Another green tea catechin, EC, is a compound characterized by high antioxidant and anti-inflammatory bioactivity [34]. Available data indicate that EC in concentrations of 0.1–1 mM have a nitrite-inhibiting activity, which in turn, is the next product of nitric oxide. EC causes apoptosis and DNA damage in acute myeloid leukemia cells in animal models of rats [34]. In addition, EC inhibits the binding of NF-κ B to Jurkat T cells and Hodgin’s lymphoma, which causes cell proliferation [35]. The disruption of proliferation of EC-treated cells can be explained by Na^+^/H^+^ ATPase inhibition [35].

Research indicates that EC affects trunsduction of signaling pathways. At micromolar concentrations, EC inhibits Er2 phosphorylation, which belongs to the Ras/MAPK pathway [34]. This pathway is essential for cellular processes such as survival and proliferation. Furthermore, EC influences NF-κ B signaling as well as induces Akt, HSP90, and eNOS phosphorylation in human HCAEC cells. In human cultured fibroblasts, EC reduces the expression of p-38 and p-JNK [36].

The available studies carried out on MDA-MB-231 and MC7 breast cancer cell cultures indicate that EGC has an inhibitory effect on the growth of cancer cells [22]. Moreover, these studies also showed that DNA damage caused by increased levels of free radicals may play a key role in the etiopathogenesis of breast cancer [22].

The inhibition of tumor cell growth was dependent on the induction of apoptosis, but without noticeable changes in the progress of the cell cycle [23]. The relationship with the p53 transcription factor is noticeable with MCF-7 cells, while MDA-MB-231 cells express the p53 mutation. Induction of apoptosis in both lines indicates the independence of apoptosis from p53 status [22]. However, the mechanism of action by EC is not fully understood and requires further research in this direction.

Epicatechin (EC) can be considered also as an otoprotective agent. EC inhibited activation of JNK, ERK, cytochrome-c and caspase-3 by cisplatin. EC may have clinical use as a chemopreventive agent that prevents cisplatin ototoxicity [37].

Research of the effect of epicatechin gallate is very limited. It is known that epicatechin gallate does not induce the expression of the gene encoding NQO1 [23]. Moreover, the ECG content of green tea varies between 3 and 6%. ECG and EGCG also have a strong inhibitory effect on the adhesion of Streptococcus mutans JC-2 bacteria [38]. Similar to other green tea catechins, ECG has an antioxidant effect, but this catechin requires further research.

## 5. Conclusions

Protein tyrosine phosphatases have recently become a potential pharmacological target for the design of new generation drugs. Protein tyrosine phosphatases PTP1B are over-expressed in breast cancer cells, trigger the growth of the tumor, and act as signaling oncogenic functions to promote growth factors and cytokines [39]. Due to the fact that PTP1B tyrosine phosphatase may be a good target for treatment, its inhibitors could be useful in a support treatment for systemic drugs as supplements. We decided to assess the inhibitory properties of green tea active compounds, not only on breast cancer cells, but also on enzymatic activity of PTP1B phosphatase involved in breast cancer development.

In our studies, we focused on the effect of four main green tea catechins described above on activity of pro-oncogenic PTP1B recombinant phosphatase as well as on viability of MCF-7 breast cancer cells. We have found that epigallocatechin, epigallocatechin gallate, epicatechin, and epicatechin gallate are able to decrease enzymatic activity of PTP1B phosphatase as well as the viability of MCF-7 cells. We discovered that from tested catechins epigallocatechin and epigallocatechin gallate were most effective inhibitors of the MCF-7 cell viability. Epigallocatechin was additionally the strongest inhibitor of PTP1B activity. We performed computational analysis of epigallocatechin binding to PTP1B. We discovered the most predicted binding pose of epigallocatechin to PTP1B.

The obtained results can be used in a future to increase the effect of anticancer systemic drugs and to avoid its oxidative side effects epigallocatechin and epigallocatechin gallate are promising agents as supplements to support the anti-cancer treatment for breast cancer.

## Figures and Tables

**Table 1:** Inhibitory activity of compounds as calculated IC_50_ values against PTP1B phosphatase enzymatic activity in comparison to the calculated IC_50_ values against MCF-7 viability.

	IC_50_ [µM]	
	PTP1B	MCF-7
EPICATECHIN	>500.0	113.2 ± 22.6
EPIGALLOCATECHIN	103.8 ± 10.1	35.9 ± 10.6
EPICATECHIN GALLATE	>500.0	>125.0
EPIGALLOCATECHIN GALLATE	>500.0	13.9 ± 3.1
